# Self-supervised learning for interventional image analytics: toward robust device trackers

**DOI:** 10.1117/1.JMI.11.3.035001

**Published:** 2024-05-15

**Authors:** Saahil Islam, Venkatesh N. Murthy, Dominik Neumann, Badhan Kumar Das, Puneet Sharma, Andreas Maier, Dorin Comaniciu, Florin C. Ghesu

**Affiliations:** a Friedrich-Alexander-Universität Erlangen-Nürnberg, Pattern Recognition Lab, Erlangen, Germany; b Siemens Healthineers, Digital Technology and Innovation, Erlangen, Germany; c Siemens Healthineers, Digital Technology and Innovation, Princeton, New Jersey, United States

**Keywords:** self-supervised learning, device tracking, interventional imaging

## Abstract

**Purpose:**

The accurate detection and tracking of devices, such as guiding catheters in live X-ray image acquisitions, are essential prerequisites for endovascular cardiac interventions. This information is leveraged for procedural guidance, e.g., directing stent placements. To ensure procedural safety and efficacy, there is a need for high robustness/no failures during tracking. To achieve this, one needs to efficiently tackle challenges, such as device obscuration by the contrast agent or other external devices or wires and changes in the field-of-view or acquisition angle, as well as the continuous movement due to cardiac and respiratory motion.

**Approach:**

To overcome the aforementioned challenges, we propose an approach to learn spatio-temporal features from a very large data cohort of over 16 million interventional X-ray frames using self-supervision for image sequence data. Our approach is based on a masked image modeling technique that leverages frame interpolation-based reconstruction to learn fine inter-frame temporal correspondences. The features encoded in the resulting model are fine-tuned downstream in a light-weight model.

**Results:**

Our approach achieves state-of-the-art performance, in particular for robustness, compared to ultra optimized reference solutions (that use multi-stage feature fusion or multi-task and flow regularization). The experiments show that our method achieves a 66.31% reduction in the maximum tracking error against the reference solutions (23.20% when flow regularization is used), achieving a success score of 97.95% at a 3× faster inference speed of 42 frames-per-second (on GPU). In addition, we achieve a 20% reduction in the standard deviation of errors, which indicates a much more stable tracking performance.

**Conclusions:**

The proposed data-driven approach achieves superior performance, particularly in robustness and speed compared with the frequently used multi-modular approaches for device tracking. The results encourage the use of our approach in various other tasks within interventional image analytics that require effective understanding of spatio-temporal semantics.

## 1 Introduction

The tracking of interventional devices is an important prerequisite for interventional specialists during catheterized cardiac interventions, such as percutaneous coronary interventions (PCIs), cardiac electrophysiology, or transarterial chemoembolization.^1^^–^^3^

Tracking the tip of the catheter as a visual guidance facilitates navigation to the desired anatomy. Furthermore, the tip of the catheter serves as an anchor point separating the catheter from the vessel structures. The anchor point can provide a basis for mapping angiography (high-dose X-ray with an injected contrast agent) to fluoroscopy (low-dose X-ray), thereby reducing the usage of contrast for visualizing vessels.^1^^,^^4^ To co-register intravascular ultrasonography with angiography and perform a complete examination of the vessel, lumen, and wall structure, catheter tip tracking also offers a significant cue.^5^^–^^7^

However, tracking the tip of the catheter in X-ray images can be challenging in the presence of various occlusions due to the contrast agent and other devices. This is in addition to the cardiac and breathing motion of the patient. Recently, self-supervised learning methods have been developed with the aim to learn general features from unlabeled data to boost the performance in various natural sequence imaging tasks. Most self-supervised pretraining methods learn such features by identifying and removing inherent redundancies from sequence image data. VideoMAE^8^ conducts temporal downsampling on the pixel level followed by symmetrical masking over all of the sampled frames with a high masking ratio of 90%. This deliberate design choice prevents the network from learning fine inter-frame correspondences. SiamMAE^9^ improves upon this baseline using highly asymmetric masking. However, the proposed asymmetric masking requires feeding in the first frame entirely with 0% masking, which increases the computation complexity quadratically and prevents the network from learning spatio-temporal features over a longer period of time.

The space-time semantics in interventional cardiac image sequences differ from natural videos in terms of both redundancies and motion. For example, visibility may largely vary based on X-ray dosage along with varying motion based on the acquisition frame-rate, patient’s breathing and cardiac motion. In angiography sequences, vessels have high structural similarity with devices, such as catheters and guidewires, and can gradually appear or disappear over time.

To address these challenges, in this work, we bring the following contributions in terms of both self-supervised pretraining and the downstream device tracking.

**Fig. 1:**
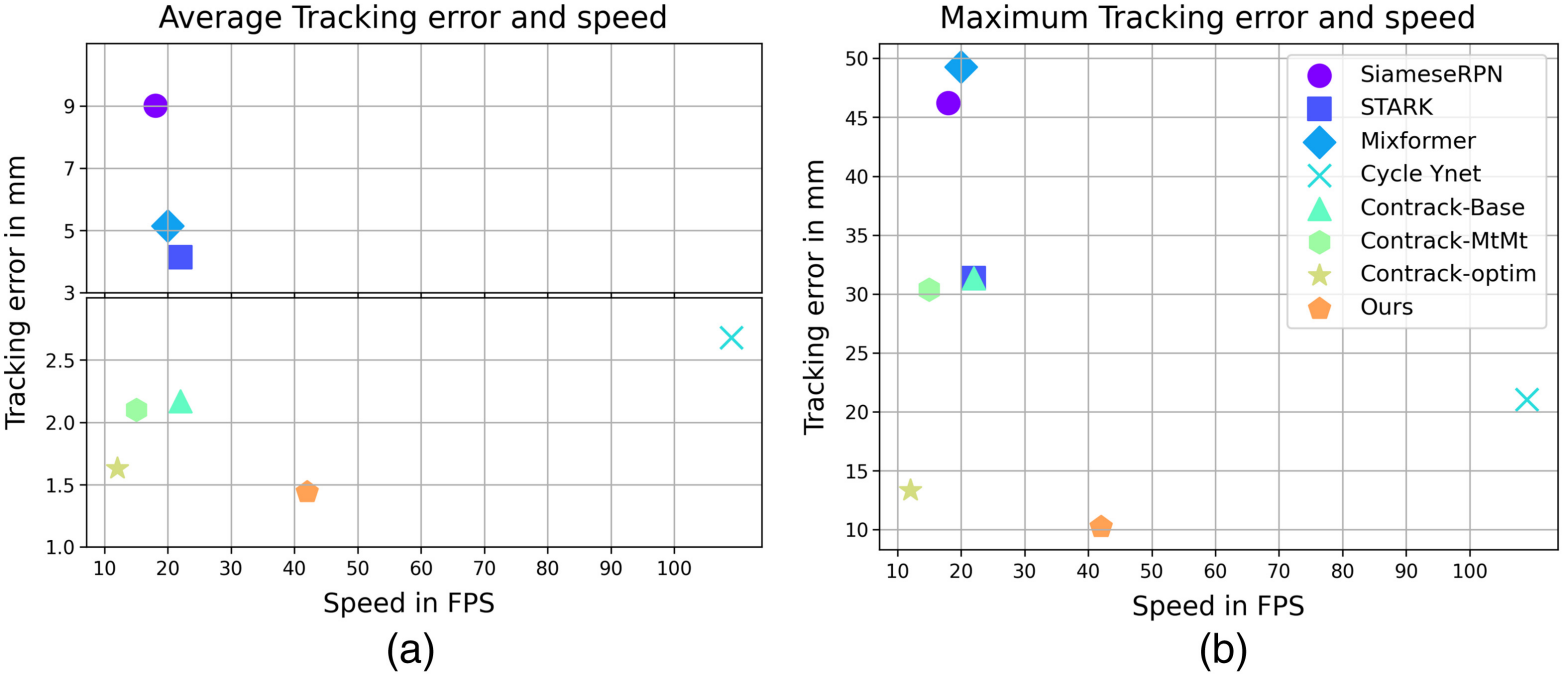
Tracking error (↓) versus average speed (↑) for catheter tip tracking in coronary X-ray sequences acquired during procedures, such as invasive coronary angiography (ICA) or PCI: (a) average tracking error and (b) maximum tracking error. Note that the average tracking error has two different scales indicated with a horizontal break-point for better visualization. The runtime is measured on a Tesla V100 GPU.

**Fig. 2:**
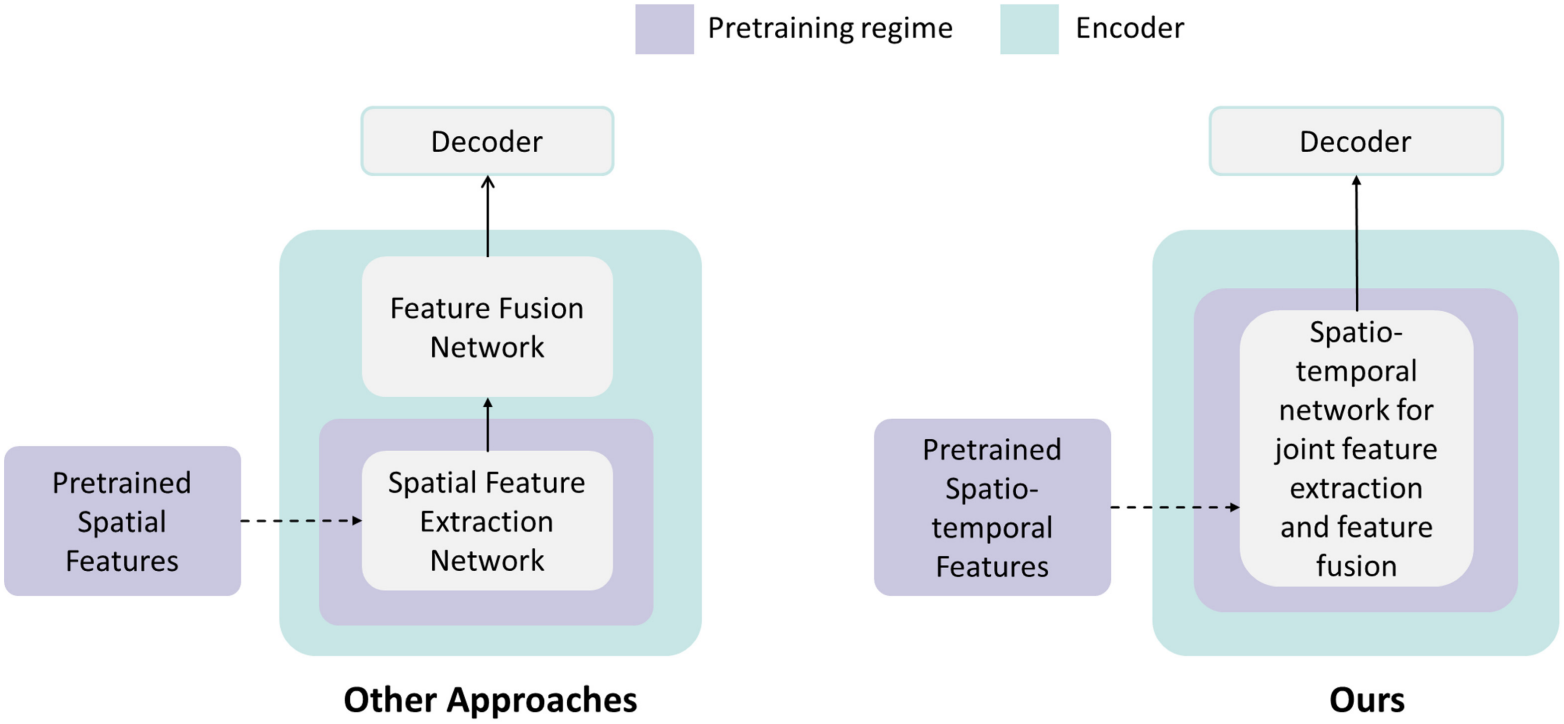
Overview of the key differences between our approach and previous approaches for device tracking.

1. We pretrain a spatio-temporal encoder on a large database of interventional cardiac X-ray sequences from over 20,000 patients (over 16,000,000 frames) for robust device tracking.
2. We propose a novel frame interpolation masked auto-encoder (FIMAE) to learn generalized spatio-temporal features from this dataset. The pretrained spatio-temporal features play an essential role in feature extraction and feature matching for tracking. Our pretrained features efficiently capture the underlying temporal motion needed for tracking, which is typically accomplished through highly optimized supplementary modules in other device tracking models.^10^^,^^11^
3. To the best of our knowledge, this is the first approach that leverages spatio-temporal pretrained features to replace a commonly used Siamese-like architecture for single object tracking.
4. A lightweight vision transformer (ViT)^12^ based model is designed to leverage the learned features to replace a traditional two-stage tracking encoder for feature extraction and feature fusion into one spatio-temporal encoder for a highly accurate and robust real-time device tracking with an inference speed of 42 fps on a single Tesla V100 GPU (refer to Figs. 1 and 2).
5. We conduct comprehensive numerical experiments and demonstrate that our method outperforms other state-of-the-art tracking methods in robustness, accuracy, and speed.
6. We conduct a comprehensive analysis of our model’s robustness in handling long temporal sequences and demonstrate its ability to maintain consistent performance across diverse scenarios, including angiography, fluoroscopy, and sequences featuring additional obstructions caused by other devices.

## 2 Related Work

### 2.1 Self-Supervised Learning

These methods have been used in a variety of contexts to learn features from unlabeled data that boost the performance in downstream tasks, such as using pretext tasks^13^^–^^15^ and contrastive learning.^16^^–^^21^ In the space of sequential image data processing (e.g., video), temporal information has been leveraged in various ways.^22^^–^^28^ However, self-supervised methods based on masked image modeling (MIM), in which the input is masked to a high percentage and fed through an encoder-decoder network to predict the missing information, have shown significant promise recently.^29^^–^^32^ Some methods use symmetrical masking on temporally downsampled video frames to reduce space-time redundancies over a long time period^8^^,^^33^. By contrast, others^9^ use asymmetrical masking to learn inter-frame correspondence between frame pairs. However, we propose a method for both reducing space-time redundancies over a long time period and learning fine inter-frame correspondence.

### 2.2 Siamese Natural Image Tracking

These strategies leverage a Siamese architecture for matching between search and target templates, in which the extracted spatial search and template features are matched via feature fusion or a similar matching module.^34^^–^^40^ With the rise of transformers, Siamese trackers have been extended to incorporate transformer-based models, such as Stark^41^ and Mixformer,^42^ among other methods cited in Refs. 43–45.

### 2.3 Historical-Trajectory-based Natural Image Tracking

These approaches leverage prompt-based methods to integrate relevant information. In particular, the temporal information is passed into the network as prompts to incorporate the historical trajectory information. ARTrack^46^ employs a decoder that receives these encodings as well as coordinates of the searched object from previous frames as spatio-temporal prompts for a trajectory proposal. Another approach, SwinTrack,^47^ uses a multi-head cross-attention decoder that leverages both the encoder output and a motion token, which represents the past object trajectory given previous bounding box predictions.

### 2.4 Device Tracking in X-Ray

Specifically for the tracking of devices in X-Ray images, multiple approaches have been proposed; these include multiple Siamese-based architectures similar to those in natural image object tracking.^34^^,^^48^ Other methods, such as Cycle Ynet,^10^ employ a semi-supervised approach to address the lack of annotated frames in the medical domain or leverage deep learning-based Bayesian filtering for catheter tip tracking.^1^ One of the most recent approaches, ConTrack,^11^ uses a Siamese architecture and a transformer-based feature fusion model. To further refine the tracking, it incorporates a RAFT^49^ model applied to catheter body masks for estimating the optical flow.

## 3 Methods

We propose a novel FIMAE approach to train a transformer model to extract spatio-temporal features based on a large internal dataset $D_{u}$. The model is designed specifically to learn inter-frame correspondences over a large number of frames. The pretrained encoder is then used as the backbone for the downstream tracking task using supervised learning on a dataset $D_{l}$ (with expert annotations). The pretraining method and the tracking pipeline are explained in the following subsections.

### 3.1 Self-supervised Model Training

#### 3.1.1 Learning space-time embeddings

Given the unlabeled dataset $D_{u}$, n frames are sampled from an arbitrary sequence $S_{k}\in D_{u}$, ∀  k>0, where $S_{k,n}=[I_{1},I_{2},\ldots ,I_{n}]$. All image frames are randomly cropped to a size of (h,w)=384×384  pixels on a sequence level (i.e., the same crop is applied to each image). Each input of size (h,w) is spatially encoded into $n\times \frac{h}{16}\times \frac{w}{16}$ tokens of dimension $D_{m}$ with no temporal downsampling.

#### 3.1.2 Masking strategy based on frame interpolation

To learn features that capture fine spatial information and fine temporal correspondences between frames, we propose a novel masking strategy based on frame interpolation that overcomes the limitation of the symmetrical tube masking proposed by VideoMAE.^8^ Recall that the VideoMAE approach is limited in capturing fine inter-frame correspondences. Traditionally, in the domain of natural imaging, the frame interpolation task^50^^,^^51^ is defined as the sum of forward warping and backward warping of any two neighboring frames (indexed by t>0), given as

$$I_{t+1}=\tau_{\theta_{1}}(I_{t})+\tau_{\theta_{2}}(I_{t+2}),$$ (1)

where $\tau_{\theta_{1}}$ denotes the forward warping operator and $\tau_{\theta_{2}}$ denotes the backward warping operator (parametrized by $\theta_{1},\theta_{2}$). However, the change of appearance in coronary vessel structures in the presence of contrast can be much more complex than natural images. Hence, a linear operation of forward and backward warping can limit the potential of the network. In our case, we reformulate this to a learning problem, seeking to optimize the parameters θ of a deep neural network to learn a combined warping operation F as

$$I_{t+1}=F_{\theta }(I_{t},I_{t+2}).$$ (2)

**Fig. 3:**
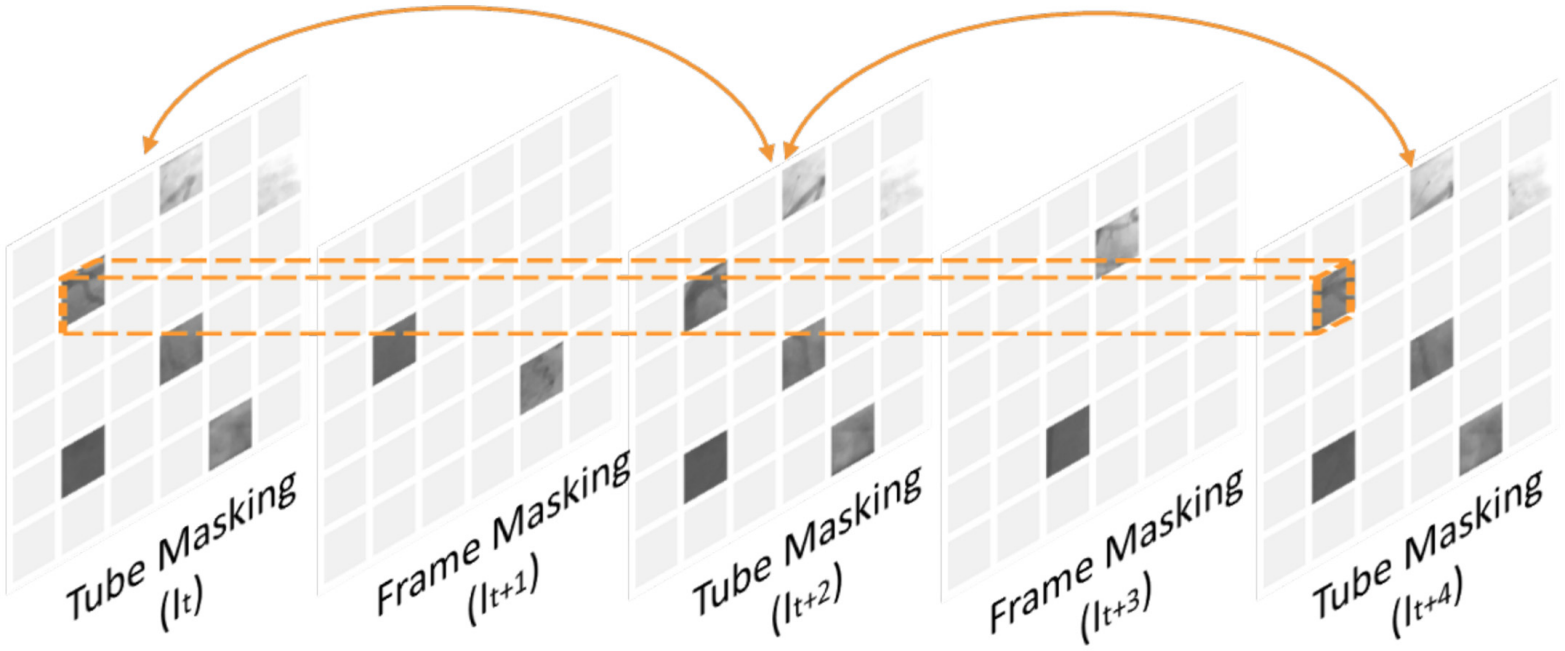
Schematic visualization of tube-frame masking.

In our approach, we use tube masking for every alternate frame with a ratio of 75% and combine it with frame masking. However, with such a high tube masking ratio, further masking an entire intermediate frame for frame interpolation can make the task extremely challenging. In addition, masking an entire frame may also lead the network to never attend to certain patch positions during training. Hence, we mask the intermediate frame randomly to a high ratio of 98%, instead. See Fig. 3 for a schematic visualization.

Let $p_{t}\in \Omega_{\text{tube}}$ be the token indices of the tube masked tokens for frame t, where $\Omega_{\text{tube}}$ denotes the set of all tube masked token indices. Similarly, $q_{t}\in \Omega_{\text{frame}}$ refers to the frame masked token indices for frame t in all randomly frame masked token indices. Mathematically, if ρ is the probability for masking, $\Omega_{\text{tube}}:\;\text{Bernoulli}(\rho_{\text{tube}})$, where different time t shares the same value. On the other hand, $\Omega_{\text{frame}}∼\text{Bernoulli}(\rho_{\text{frame}})$ and is drawn uniquely for each frame at t. Let $p_{t}^{\prime }\in \Omega_{\text{tube}}^{\prime }$ and $q_{t}^{\prime }\in \Omega_{\text{frame}}^{\prime }$ be the sets of remaining visible token indices. Combining tube and frame masking strategies, we obtain the following reconstruction objective for any three given frames:

$$I_{t},I_{t+1},I_{t+2}=F_{\theta }(I_{t}(p_{t}^{\prime }),I_{t+1}(q_{t+1}^{\prime }),I_{t+2}(p_{t+2}^{\prime })),$$ (3)

where 0<t<n−1 denotes the index of an arbitrary frame from the sampled sequence and $I_{t}(p_{t}^{\prime })$ denotes the visible patches of frame $I_{t}$ with tube/frame masking applied. The three-frame objective shown in Eq. (3) can be generalized to all n frames.

#### 3.1.3 Encoder-decoder training

The unmasked patches are passed through a ViT encoder, which adopts a joint space-time attention, that is, each token for frame t, is projected and flattened into $D_{m}$-dimensional vector query, key, and value embedding: $\left(q_{t},k_{t},v_{t}\right)$. The joint space-time attention is based on the concatenated vectors, given as

$$\text{Attention}(Q,K,V)=\text{softmax}(\frac{QK^{T}}{\sqrt{d}})V,$$ (4)

where the variables (Q,K,V) are defined as $Q=\text{Concat}(q_{1},q_{2},\ldots ,q_{n})$, $K=\text{Concat}(k_{1},k_{2},\ldots ,k_{n})$, $V=\text{Concat}(v_{1},v_{2},\ldots ,v_{n})$ for n sampled consecutive frames. The encoded visible patches are then concatenated with learnable masked tokens. A lightweight transformer decoder attends to the encoded patches and the masked token to reconstruct the initially masked patches. The decoder incorporates additional positional encoding to ensure the correct positions of the masked and unmasked patches as per the original frames.

#### 3.1.4 Pretraining loss function

We use a weighted mean squared error loss, $L=L_{\text{tube}}+\gamma L_{\text{frame}}$ between the masked tokens and the reconstructed ones in the pixel space based on the masking strategy, where γ is the weighting factor. The losses are calculated as

Ltube=1|Ωtube|∑t=2η+1n∑pt∈Ωtube‖It(pt)−I^t(pt)‖2, (5)

Lframe=1|Ωframe|∑t=2η+2n∑qt∈Ωframe‖It(qt)−I^t(qt)‖2, (6)

where I is the input image, $\hat{I}$ is the reconstructed image, and 0≤η≤(n−2)/2. We use a weighted loss for reconstruction to compensate for the imbalance between low masked frames (less reconstruction tokens) and highly masked frames (more reconstruction tokens). The variable γ is defined as the ratio of the number of $\Omega_{\text{tube}}$ tokens and the number of $\Omega_{\text{frame}}$ tokens.

### 3.2 Downstream Application: Device Tracking

In particular, for tracking the tip of the catheter, our goal is to track its location, ${\hat{y}}_{t}=(u_{t},v_{t})$ at any time t,t>0 given a sequence of X-ray images ${\left\{I_{t}\right\}}_{t=1}^{n}$ with a known initial location of the catheter tip $y_{1}=(u_{1},v_{1})$ on the labeled dataset $D_{l}$. We consider the sequences $S_{k}\in D_{l}$, ∀  k>0 to have only a few annotated labels, $S_{k,n}=[(I_{1},y_{1}),(I_{2}),\ldots ,(I_{7},y_{7}),(I_{8}),\ldots ]$. To identify the location of the tip of the catheter at the current search frame, existing approaches build a correlation with a template frame. The template frame is usually a small crop around the catheter tip location from a previously predicted frame. Similar to ConTrack, during training, we use three template frames that are cropped from the first annotated frame and the previous two annotated frames, respectively. We use the current frame for template frames if no previously annotated frames are available. During inference, the initial location of the catheter tip serves as the first template crop and is kept intact. The remaining two template frames are updated dynamically based on the model’s predictions.

#### 3.2.1 Feature transfer

**Fig. 4:**
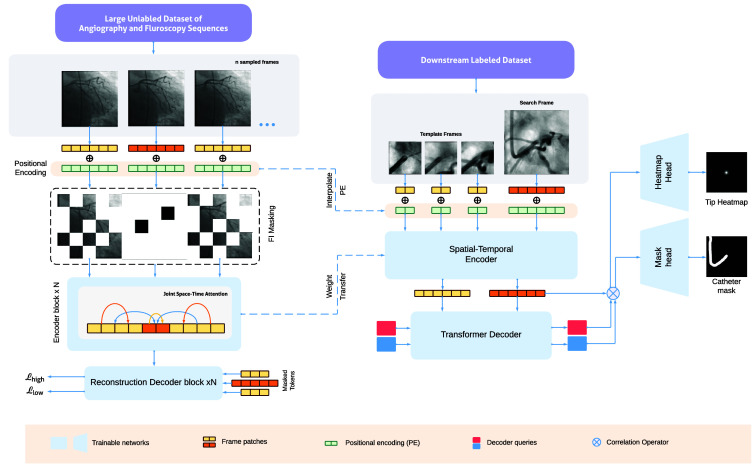
Overview of our framework. First, the encoder is trained to learn spatio-temporal features from a large unlabeled dataset of angiography and fluorscopy using FIMAE (a). Then, the weights are transfered into the ViT encoder for feature extraction and feature matching for tracking the catheter tip (b) (Video 1, MP4, 40.3 MB [URL: https://doi.org/10.1117/1.JMI.11.3.035001.s1]).

The spatio-temporal transformer backbone inputs three template frames and a search frame as four distinct frames. We interpolate the positional encoding from the pretraining frame positions appropriately to ensure that the network distinguishes each template and search frame as distinct frames. In particular, each template frame and the search frame correspond to the positions of center crops of individual frames in the pretraining setup. Therefore, the encoder inputs $\text{Concat}(te_{1},te_{2},te_{3},se)$, where $te_{1,2,3}$ and se are template patches and search patches, respectively. Given that transformers are isotropic models, we obtain an encoded feature set, $f_{c}=\text{Concat}(f_{te_{1}},f_{te_{2}},f_{te_{3}},f_{se})$. The spatio-temporal transformer backbone is trained to extract fine inter-frame correspondences. Hence, this results in a joint feature extraction and feature matching between the template frames and the search frame. The overview of the proposed model is depicted in Fig. 4.

#### 3.2.2 Multi-task transformer decoder

We use a lightweight transformer decoder similar to the original transformer model.^52^ First, all of the features $f_{c}$ are projected to a lower dimension $d_{m}$. The decoder uses two learnable query tokens $\left(h_{d},m_{d}\right)$, one for a heatmap head and one for a mask head. Then, each layer first computes attention on the query tokens as per Eq. (4). It is followed by cross-attention with encoded features $f_{c}$, where key and value embeddings are computed by projecting the features $f_{c}$ to dimension $d_{m}$. The resulting query tokens are then correlated with the search features, unflattened, and passed through a convolutional neural network (CNN) head. The catheter predicted heatmap and mask are given as

$$P_{h}={\mathrm{Conv}}_{h}(\text{Unflatten}(\mathrm{corr}(f_{se},h_{d}))),$$ (7)

$$P_{m}={\mathrm{Conv}}_{m}(\text{Unflatten}(\mathrm{corr}(f_{se},m_{d}))).$$ (8)

The final tip coordinates are obtained by $\hat{y}=\max(P_{h})$, where $P_{h}$ and $P_{m}$ refer to the predicted heatmap of the catheter tip and predicted mask of the catheter, respectively. We compute soft dice loss $L_{\text{dice}}=L_{h}+\lambda L_{m}$, for both heatmap and mask predictions, given as

$$L_{h}=\frac{2*\sum G_{h}*P_{h}}{\sum G_{h}^{2}+\sum P_{h}^{2}+\epsilon },$$ (9)

$$L_{m}=\{\begin{array}{ll} \frac{2*\sum G_{m}*P_{m}}{\sum G_{m}^{2}+\sum P_{m}^{2}+\epsilon }, & \text{if}\;G_{m}\;\text{exists}\\ 0 & \text{otherwise}, \end{array}$$ (10)

where G represents ground truth labels and λ is the weight for the weighting mask loss.

## 4 Experiments and Results

### 4.1 Dataset

An unlabeled internal dataset $D_{u}$ of coronary X-ray sequences is utilized to pretrain our model. $D_{u}$ consists of 241,362 sequences collected from 21,589 patients, comprising 16,342,992 frames in total. It contains both fluoroscopy (“Fluoro”) and angiography (“Angio”) sequences. We randomly sample 10 frames at a time, with varying temporal gaps between them, ranging from 1 to 4 frames. We repeat the last frame in sequences in which the number of frames is less than 10. The model is then pretrained for 200 epochs with a learning rate of $1e^{-4}$.

**Fig. 5:**
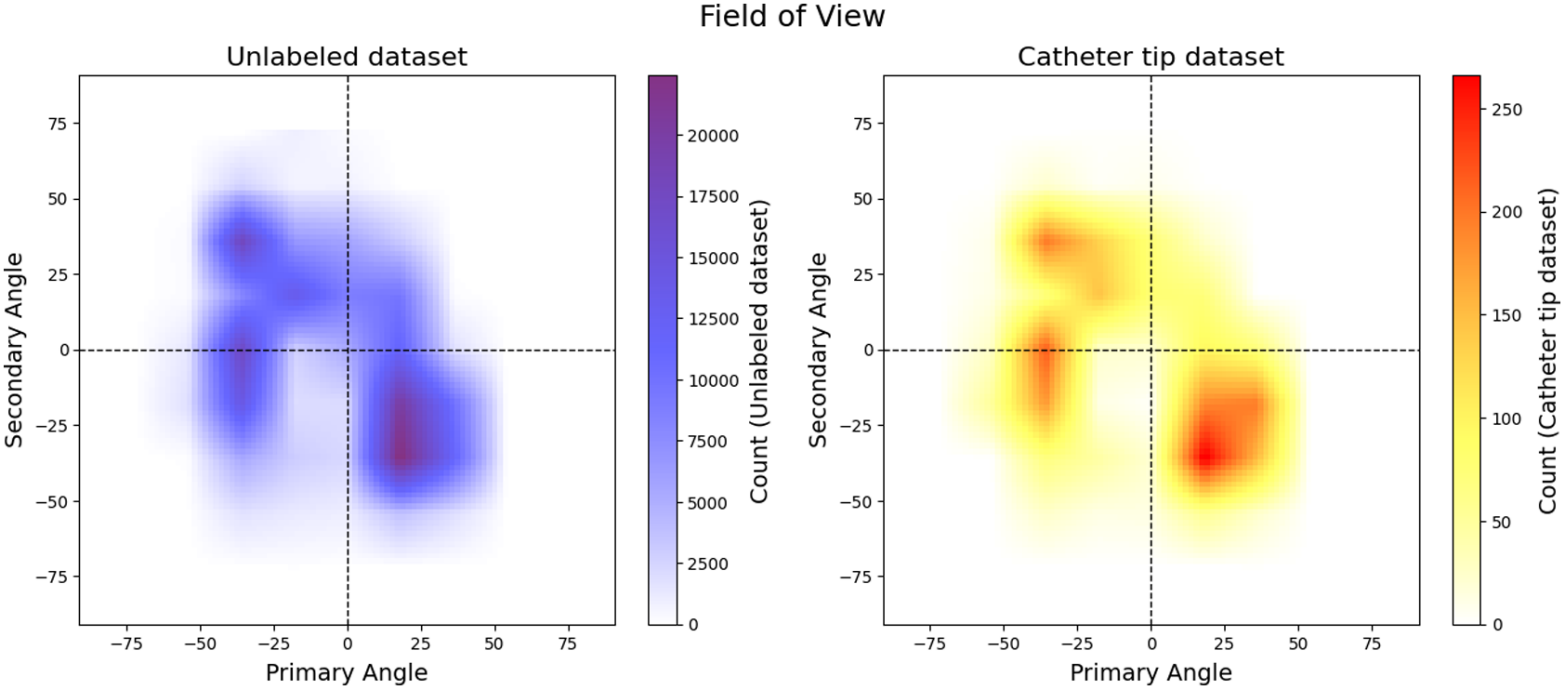
Distribution of the datasets based on the field of view (positioner primary angle and positioner secondary angle): the left plot denotes the unlabeled dataset ($D_{u}$) and the right plot denotes the catheter tip dataset ($D_{l}$).

For the downstream tracking task, we use dataset $D_{l}$. Note that $D_{l}\cap D_{u}=Ø$. The distribution of the field of view for both $D_{u}$ and $D_{l}$ is depicted in Fig. 5 and is estimated based on the positioner angles. The positioner primary angle is defined in the transaxial plane at the imaging device’s isocenter with zero degrees in the direction perpendicular to the patient’s chest, +90  deg at the patient’s left side, and −90 at the patient’s right side. The positioner secondary angle is defined in the sagittal plane at the imaging device’s isocenter with zero degrees in the direction perpendicular to the patient’s chest. Figure 5 shows that the distribution of the sequences in both datasets are concentrated around similar positioner angles. Other attributes from both datasets $D_{l}$ and $D_{u}$ are depicted in Table 1.

**Table 1:** Dataset statistics (range and median) for unlabeled dataset ($D_{u}$) and Catheter tip dataset ($D_{l}$).

	Unlabeled dataset ($D_{u}$)		Catheter tip dataset ($D_{l}$)	
Attributes	Range	Median	Range	Median
FPS	1 to 30	15	1 to 30	15
No. of frames	1 to 552	83	4 to 920	77
Resolution (mm/pixel)	0.129 to 0.616	0.279	0.108 to 0.368	0.279
Peak kilo volt	45.16 to 125.0	87.1	61.0 to 125.0	86.3
Tube current (mA)	1.0 to 928.0	757.0	7.0 to 904.0	740.0
Exposure time (msec)	3 to 20235	522	5 to 14160	503

**Fig. 6:**
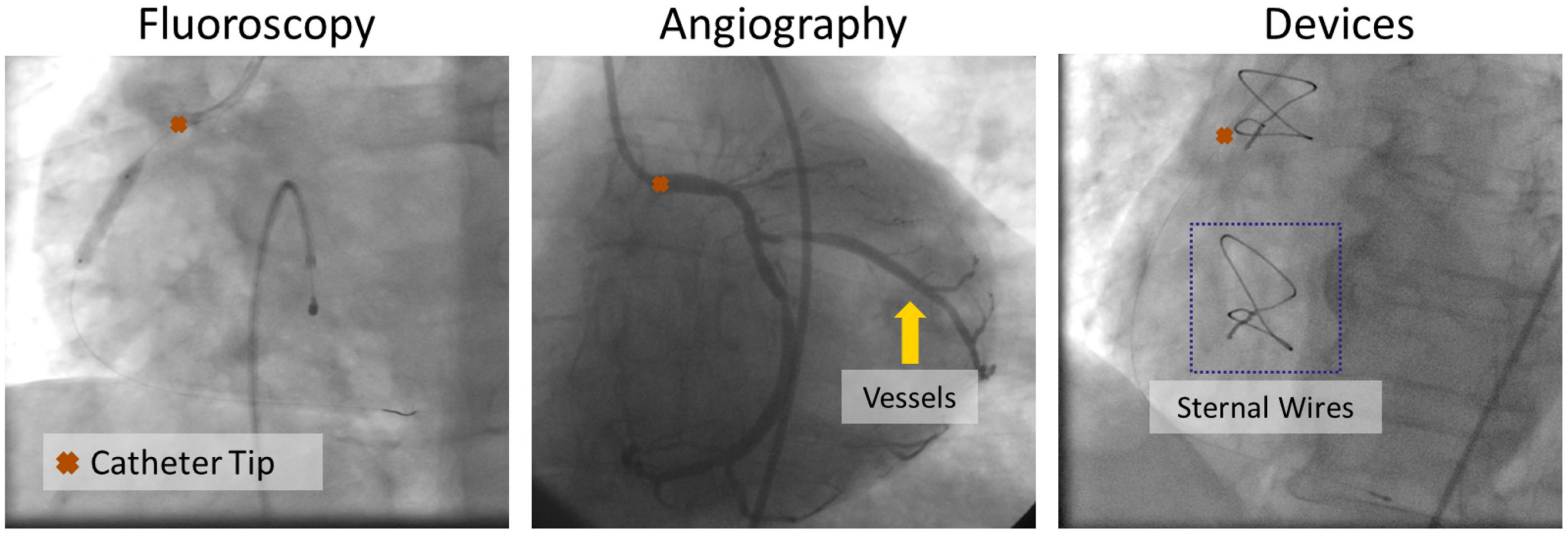
Visualization of tip of the catheter in fluoroscopy, angiography, and cases with other devices.

The annotations on the frames in $D_{l}$ represent the coordinates of the tip of the catheter, which are converted to Gaussian heatmaps with standard deviations of ≈5  mm. Mask annotations of the catheter body are also available for a subset of these annotated frames. On average, the catheter body takes up 0.009% of the total area of a frame. The training and validation set consists of 2314 sequences totaling 198,993 frames, out of which 44,957 have annotations. In this set, 2,098 sequences are Angio and only 216 sequences are Fluoro. The test set consists of 219 sequences, in which all 17,988 frames are annotated. For evaluation, we split the test set into three categories: 94 Fluoro sequences (8494 frames and 82 patients), 101 Angio sequences (6904 frames and 81 patients), and 24 devices sequences (2593 frames and 10 patients).^11^ The latter category, “devices,” covers all sequences in which sternal wires are present; these cause occlusion and thus further increase the difficulty of catheter tip tracking. Examples of these cases are illustrated in Fig. 6. The signal to noise ratio (SNR) of the image intensity at the catheter tip with respect to the background is shown in Table 2, further quantifying the challenge of tracking. The SNR was calculated based on the following equation:

$$\mathrm{SNR}=20\;\log_{10}\;\frac{P_{w}}{\sigma_{f}},$$ (11)

where $P_{w}$ is the mean intensity in the window of size 6×6 (≈2  mm×2  mm) and $\sigma_{f}$ denotes the standard deviation of the intensity of the background in the window of size 30×30 (≈10  mm×10  mm) with the catheter tip as the center of both windows.

**Table 2:** SNR of different categories in the catheter tip dataset ($D_{l}$).

Fluoro	Angio	Devices
24.72 dB	21.38 dB	23.64 dB

We follow the same image pre-processing pipeline as ConTrack, i.e., we resample and pad to the size of 512×512 with 0.308 mm isotropic pixel spacing. We use 160×160 crops for the search image and 64×64 crops for the template images. We train our model for 100 epochs, with a learning rate of $2e^{-4}$ using AdamW optimizer and cosine annealing scheduler with warm restarts.

### 4.2 Performance Evaluation

We evaluate our work against state-of-the-art methods, explore the impact of the proposed pretraining strategy, and investigate whether complex additional tracking refinement modules are necessary. All of the evaluations are performed based on expert annotations.

#### 4.2.1 Benchmarking against state-of-the-art

**Fig. 7:**
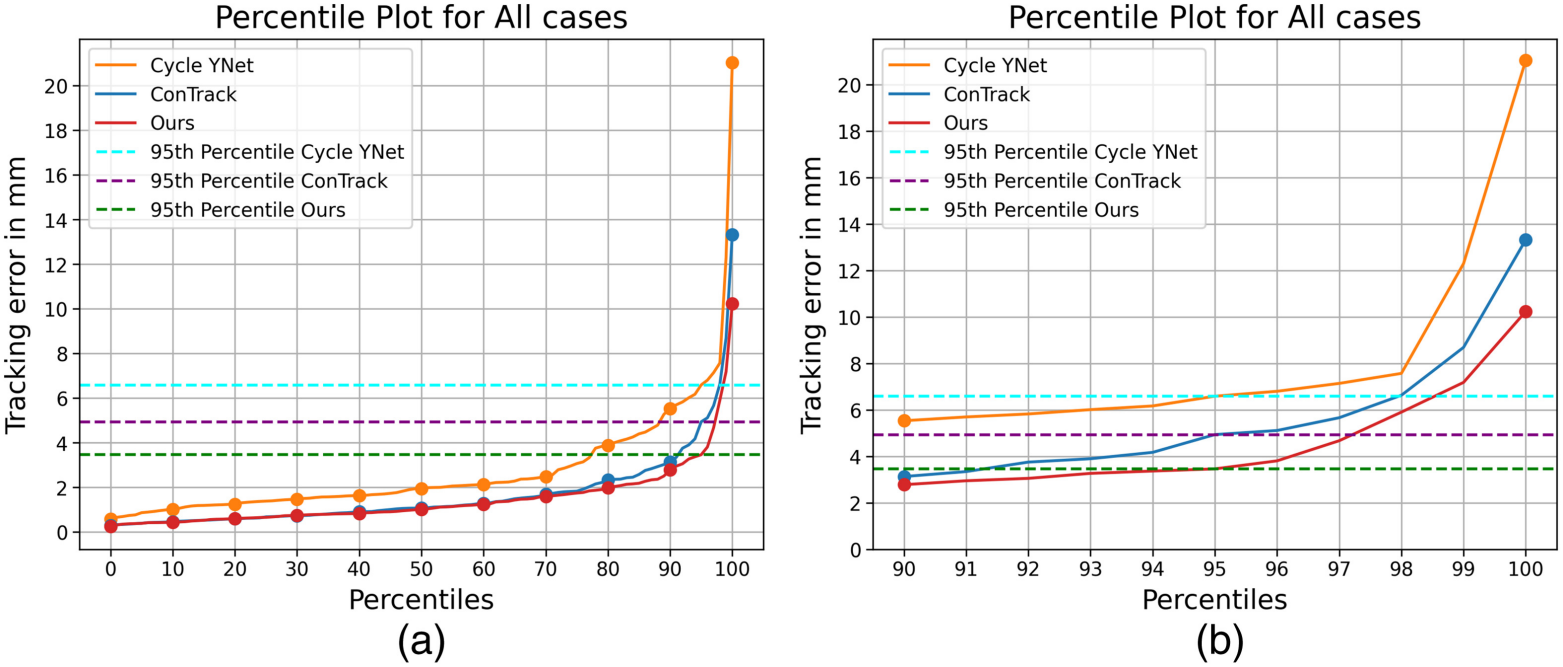
Percentile plot of Cycle YNet, ConTrack, and Ours (a) for all test cases and (b) zoomed in for percentiles from 90’th to 100’th. The 95’th percentile of our method’s performance is lesser than the average diameter of the vessels (≈4  mm).

We report the performance of our model against the state of the art device tracking models in Table 3. Here, we evaluate the euclidean distance error in mm between the prediction and the ground truth annotations. Overall, our method demonstrates the best performance on the test dataset, excelling in both precision and robustness. Our approach significantly reduces the overall maximum error, e.g., by 66.31% against the comparable version of ConTrack (ConTrack-mtmt) and by 23.20% against ConTrack-optim, a highly optimized solution leveraging multi-stage feature fusion, multi-task learning, and flow regularization. In comparison with previous state-of-the-art approaches, our approach results in fewer failures, as depicted by the error distribution in Fig. 7. At least 95% of all test cases has an error below the average diameter of the vessels (≈4  mm). Notably, our approach stands out from other tracking models by eliminating the need for a two-stage process involving the extraction of spatial features and subsequent matching using feature fusion. Instead, our spatio-temporal encoder jointly performs both.

**Table 3:** Comparison study of sequence-level tracking errors (mean euclidean distance) and runtime for different methods for catheter tip tracking in coronary X-ray sequences. The best numbers are marked in bold. We also show the performance of different versions of ConTrack. ConTrack-base refers to its base version, which has no additional modules; ConTrack-mtmt refers to multi-task and multi-template version; and ConTrack-optim is its final optimal version, which has all modules including flow refinement.

Models	Median (mm) ↓	Mean (mm) ↓	Std (mm) ↓	95 percentile (mm) ↓	Max (mm) ↓	Speed (fps) ↑
SiameseRPN^34^	7.13	9.01	6.81	22.37	46.23	18
STARK^41^	2.65	4.14	4.93	9.24	31.34	22
MixFormer^42^	2.68	5.15	7.1	19.20	49.29	20
Cycle Ynet^10^	1.96	2.68	2.4	6.75	21.04	**109**
ConTrack-base^11^	1.13	2.17	3.75	6.34	31.35	21
ConTrack-mtmt^11^	1.12	1.97	3.61	5.53	30.37	19
ConTrack-optim^11^	1.08	1.63	1.7	5.18	13.32	12
Ours	**1.02**	**1.44**	**1.35**	**3.52**	**10.23**	42

Other approaches often require two or more forward passes for two-stage processing to incorporate varying the template-search size, which increases computational complexity. This is further amplified by the inclusion of additional modules, such as multi-task decoders and the flow-refinement network in ConTrack-optim.^11^ By contrast, our model accomplishes the task with a single forward pass for both the multiple templates and the search frame. The only additional modules in our model are the two CNN heads for multi-task decoding. This design choice enables us to achieve a significantly higher real-time inference speed of 42 fps on a single Tesla V100 GPU without compromising on accuracy, as shown in Fig. 1. Despite Cycle Ynet^10^ also relying on multiple forward passes for feature extraction, its simplicity and computationally friendly CNN architecture allows it to reach a higher speed, albeit at the expense of accuracy and robustness.

#### 4.2.2 Impact of pretraining

**Fig. 8:**
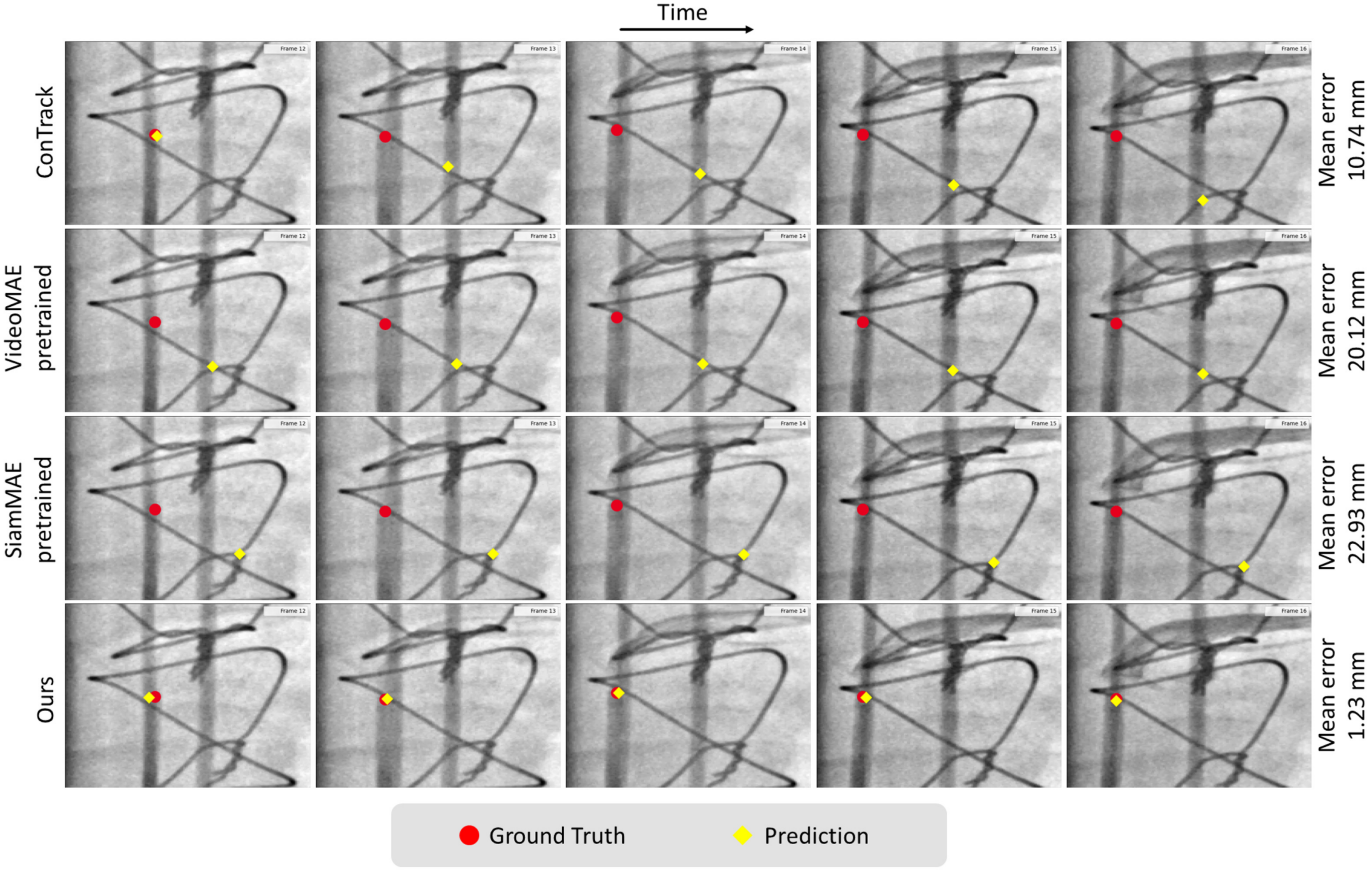
Qualitative results. Comparison of different methods on a challenging sequence of angiography, in which tracking receives obstruction from vessels and sternal wires (other devices). Note that the images have been cropped around the region of interest for better visualization. The mean error depicted in the figure is the average error computed over the entire sequence.

Next, we focused on the impact of pretraining by comparing tracking performance utilizing our proposed pretraining strategy (FIMAE) against current prevalent pretraining methods for sequential image processing; see Table 4. The findings indicate that pretraining on domain-specific data, as opposed to natural images (VideoMAE-Kinetics), offers significant advantages. However, even when including the models trained on $D_{u}$ (VideoMAE and SiamMAE) into the comparison, our model surpasses all by more than 30% across all reported metrics. VideoMAE lacks fine temporal correspondence between frames, leading to non-efficient feature matching between the template and search frames. Although SiamMAE has the ability to learn inter-frame correspondence, it relies on only two frames at a time, which is insufficient for fully capturing the underlying motion. Qualitative results, shown in Fig. 8, are based on a challenging angiography sequence with contrast-based device obstruction and other visible sternal wires. The figure shows how our model is able to handle this challenging case by not losing track of the tip of the catheter, whereas the other models fail to differentiate the catheter from the sternal wires.

**Table 4:** Study of effect of pretraining startegies on the performance of the catheter tip tracking. Pretraining is performed either on our internal dataset (denoted as $D_{u}$) or on natural images (in case of the first approach). The best values are marked in bold.

PretrainingStrategy	Median(mm)	Mean(mm)	Std(mm)	Max(mm)
VideoMAE-Kinetics	1.93	3.67	4.95	36.99
VideoMAE ($D_{u}$)	1.48	2.75	4.64	53.26
SiamMAE ($D_{u}$)	1.54	2.79	3.44	23.76
Ours: FIMAE ($D_{u}$)	**1.02**	**1.44**	**1.35**	**10.23**

#### 4.2.3 Performance without complexity

**Fig. 9:**
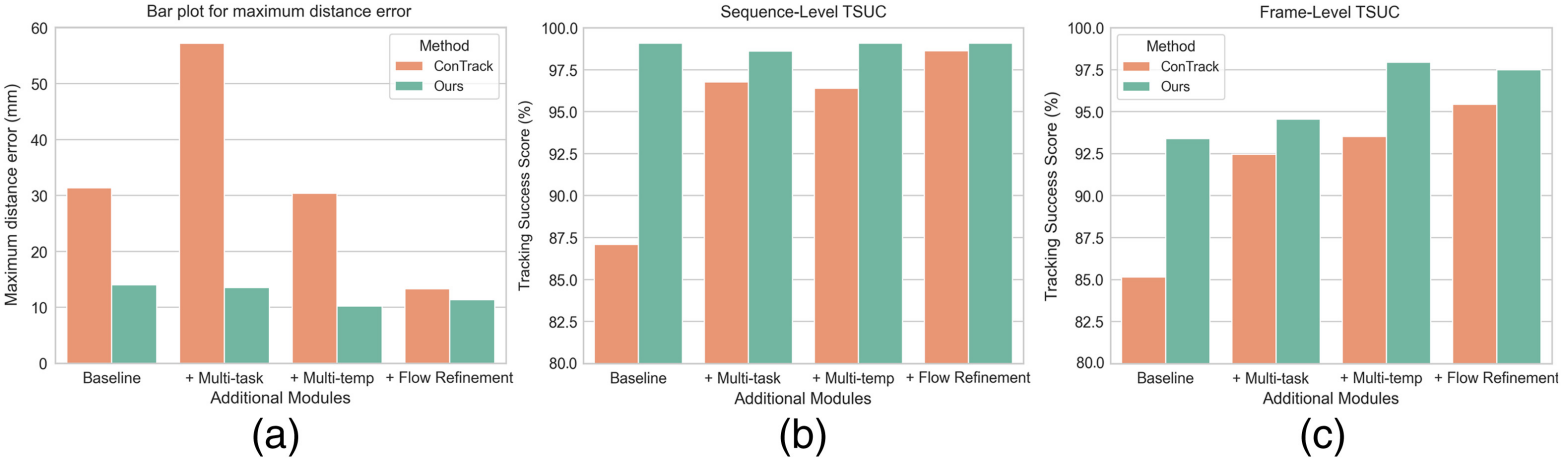
Comparison of robustness between our method and different versions of Contrack, via (a) maximum distance error (↓), (b) sequence-level TSUC (↑), and (c) frame-level TSUC (↑).

The strength of our approach comes from the pretrained spatio-temporal features that facilitate effective feature matching between the template frames and the search frame. Another key advantage is its prior understanding of the inherent cardiac/respiratory motion. This knowledge significantly reduces or even eliminates the impact of additional modules, such as flow refinement. Our approach thereby achieves high robustness in tracking, with minimal variations across different additional modules, such as multi-task. To illustrate this, Fig. 9(a) highlights the relative stability of the maximum error across different versions of our model compared with the high volatility observed in ConTrack under different module configurations. In addition, ConTrack reaches its best performance only when utilizing all modules, in particular, including flow-refinement, which in turn leads to increased inference time. Contrary to ConTrack, adding the flow refinement module to our model even reduced its performance marginally in terms of accuracy (1.54 mm) and robustness (max error of 11.38 mm). We postulate that this is attributable to the fact that, although flow refinement can indeed learn intricate temporal correspondences between the previous and current frames, it can also propagate noise originating from inaccurately predicted catheter masks.

To further assess the robustness of the tracking systems, we introduce the tracking success score (TSUC), which draws parallels with most tracking benchmarks prevalent in single object tracking in the natural image domain.^53^ TSUC is computed as the ratio of the number of instances (frame or sequence) in which the distance error falls below a specific threshold to the total number of instances. To establish a relevant threshold, we set it at twice the average vessel diameter in our test dataset (≈8  mm). Figures 9(b) and 9(c) summarize the results for sequence-level and frame-level TSUC, respectively. Our approach consistently achieves an impressive 99.08% sequence-level TSUC across all additional modules, with only a small drop to 98.61% in the multi-task configuration. At the frame level, our optimal version (multi-task multi-template) yields a TSUC of 97.95%, compared with 93.53% for ConTrack under the same configuration. ConTrack achieves its best frame-level TSUC of 95.44% using the flow-refinement variant.

**Fig. 10:**
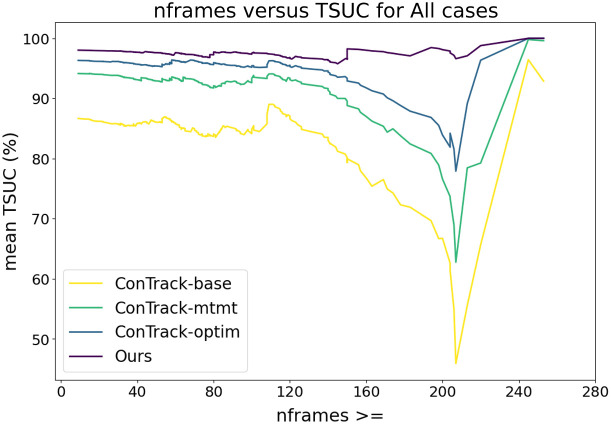
Robustness with respect to the sequence length: mean TSUC for all sequences greater than the frame count (nframes). Note that the dataset consists of only four sequences with a frame count greater than 210.

The robustness of a method is also influenced by its ability to effectively handle long sequences as the accuracy of current frame predictions is dependent on previous frame predictions, resulting in a gradual accumulation of errors over time. We examine the mean TSUC for sequences exceeding a certain frame count (nframes) in Fig. 10. The plot shows that our method consistently demonstrates stable TSUC values across various sequence lengths, indicating its robust performance. Conversely, different versions of the ConTrack exhibit a gradual decline in mean TSUC as the frame count threshold increases, suggesting a reduced reliability in predicting outcomes over extended sequences.

#### 4.2.4 Performance breakdown for different cases

**Fig. 11:**
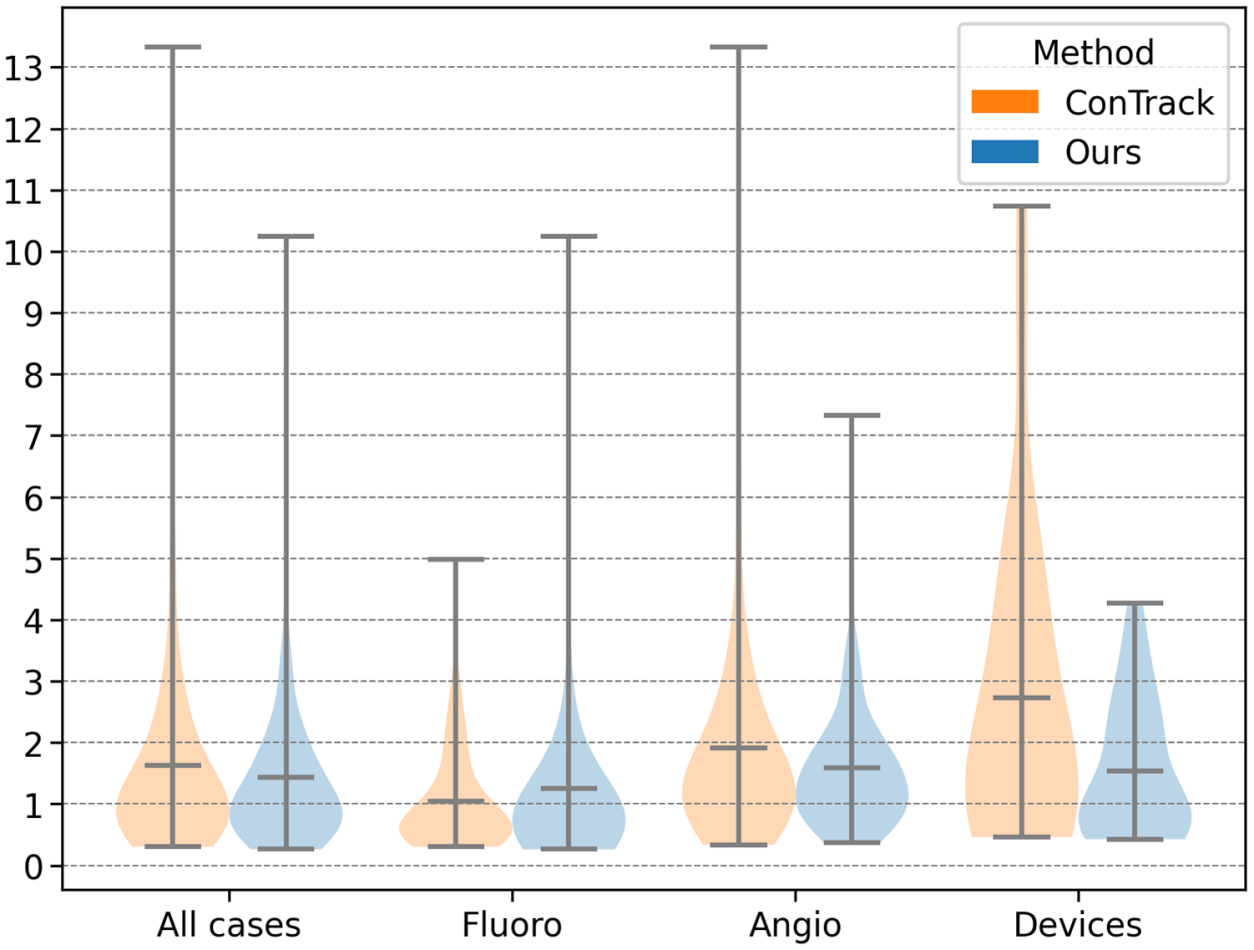
Breakdown of different cases in a violin plot for comparison of our method with ConTrack.

**Fig. 12:**
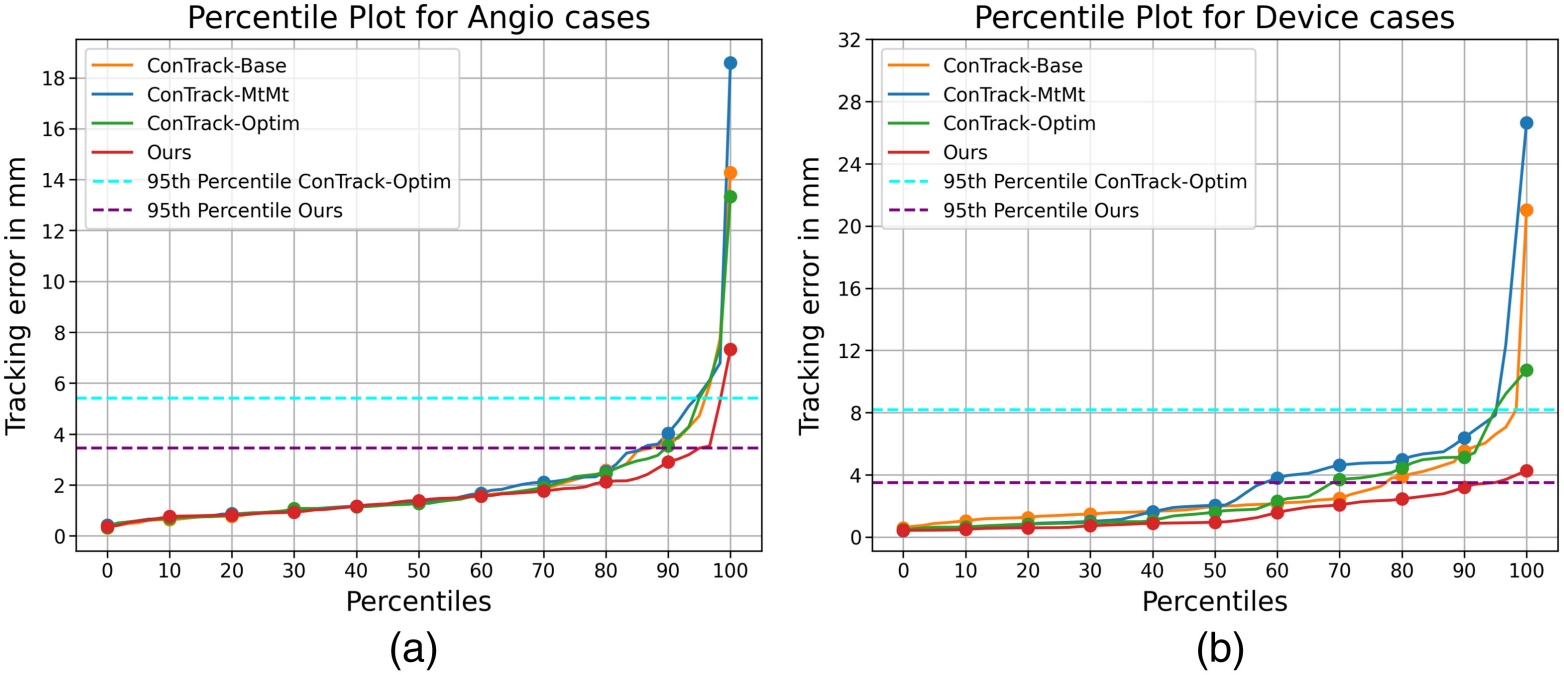
Percentile plots of different versions of ConTrack and ours for (a) Angio cases and (b) device cases.

**Fig. 13:**
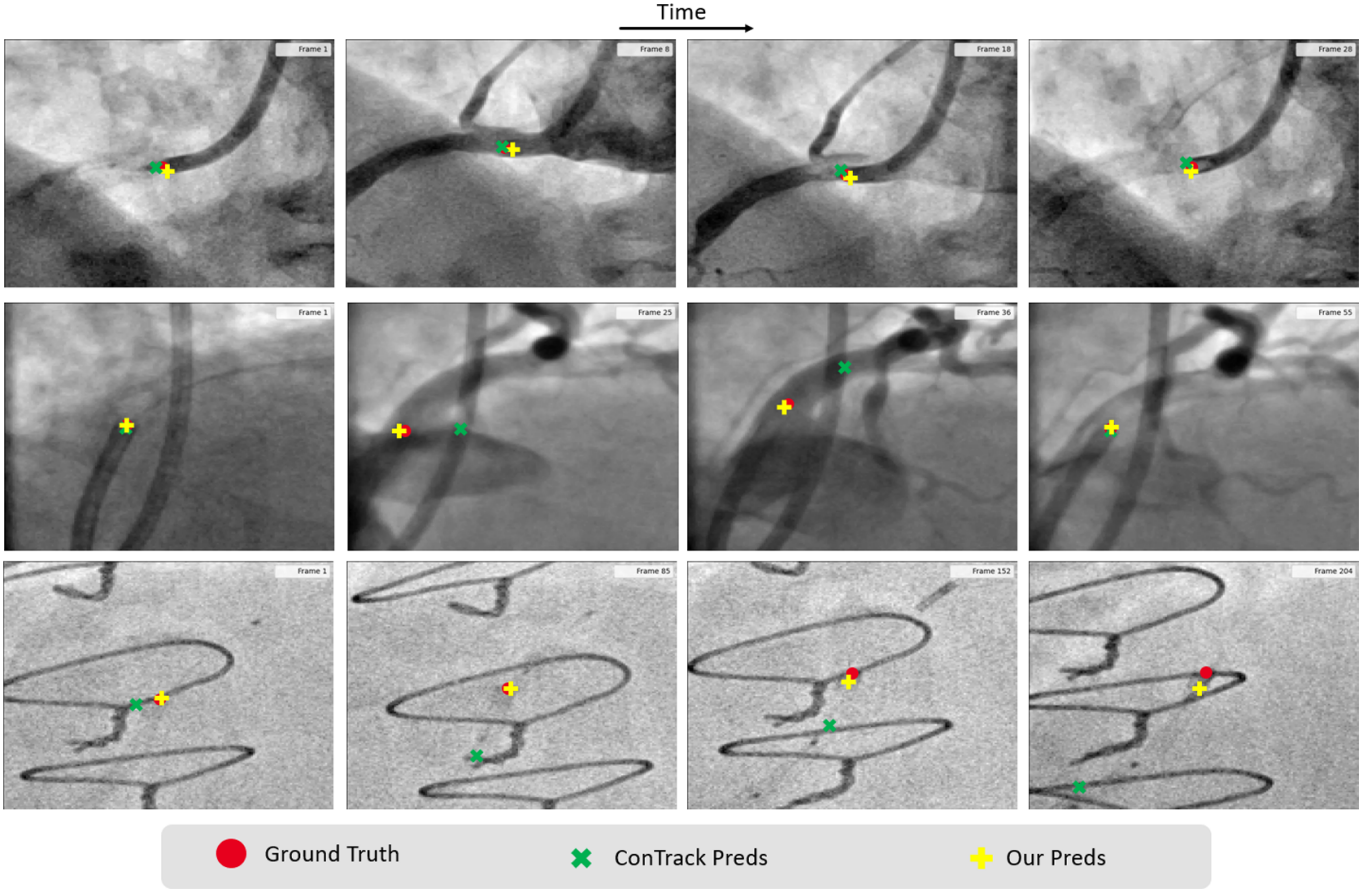
Visualization of predictions of ConTrack and our model in two Angio sequences (top two) and an extra device case (bottom). Note that the frames are sampled randomly from the sequence for visualization.

We further conduct detailed comparison with the best-performing state-of-the-art method, ConTrack, for the different image categories defined earlier; see Fig. 11. We further compare our model’s performance with ConTrack for the challenging cases, i.e., angiography and devices, via percentile plots in Fig. 12. In the cases of angiography, our method shows a 15% improved accuracy and 45% reduction in the maximum error. Similarly, for the devices (occlusion) category, we achieve a 43% better accuracy and 60% reduction in the maximum error (Figs. 11 and 12). Our model’s performance on Angio and devices cases is compared qualitatively with ConTrack in Fig. 13. The example cases in the figure show the effectiveness of our approach in the presence of complex occlusions from the vessels and sternal wires. ConTrack achieves a better performance than our method in Fluoro cases with a slightly better median and lesser maximum error. However, for Fluoro, ConTrack achieves a TSUC of 99.01% (inaccurate in one sequence) compared with our model’s TSUC of 97.69% (inaccurate in three sequences). The inaccuracy of our model is seen in sequences in which the visibility of the catheter is faint due to low-dose X-rays. We hypothesise that this is due to the transformer’s architecture using 16×16 non-overlapping patches, which makes it less effective toward faint visibility in low-dose X-rays compared with CNNs in ConTrack, which uses overlapping 3×3 windows.

### 4.3 Ablations

The following ablation studies investigate the impact of three key components on the overall tracking performance.

#### 4.3.1 Positional encoding

As reported in Table 5, the positional encoding strategy has a notable impact on the downstream task performance. The naive positional encoding simply applies 1D sine-cosine positional encoding over all patches and hence loses the temporal information about the patches, resulting in unsatisfactory results. If learnable positional encoding is used, the temporal positions are still needed to be learned, leading to sub-optimal performance. Interpolating from the central patch positions of the pretrained frames (frame-aware positional encoding) gives the best results.

**Table 5:** Effect of different positional encoding incorporated in the downstream task. The best values are marked in bold.

Positional encoding	Median	Mean	Std	Max
Naive	1.47	2.51	3.43	36.24
Learnable	1.37	1.86	1.54	11.22
Frame-aware (Ours)	**1.02**	**1.44**	**1.35**	**10.23**

#### 4.3.2 Masking ratio

We further compare the performance of different intermediate frame masking ratios in Table 6. The best results are obtained with an intermediate frame masking ratio of 98%. Although results with 95% are largely equivalent, there is a notable reduction in performance when the entire frame is masked, which may be due to the lack of patches and its relative position information during pretraining.

**Table 6:** Tracking performance with FIMAE trained with different intermediate frame masking ratios, i.e., masking ratio of $\Omega_{\text{frame}}$. The best values are marked in bold.

Frame masking ratio (%)	Median	Mean	Std	Max
95	1.09	1.47	**1.24**	10.34
98	**1.02**	**1.44**	1.35	**10.23**
100	1.08	1.78	2.09	15.12

#### 4.3.3 Effect of initialization

Recall that the first template crop during both training and inference was obtained from the initial catheter tip location and was not updated. We explore its impact in Table 7. To assess its importance, we conduct two experiments. First, we dynamically update the initial template frame during inference, as with the others. Second, we introduce random noise (2 to 16 pixels) to the initial tip location instead of updating the template. Our findings highlight the crucial role of initialization in tracking. Updating the initial template frame worsens performance due to greater accumulated prediction errors over time compared with the original setup. Additionally, even small noise levels of 2 pixels can noticeably affect performance, increasing the maximum error by 5 pixels.

**Table 7:** Significance of initialization in catheter tip tracking: how the performance is affected if first template frame is updated or some noise is introduced to the initial tip coordinates. The best values are marked in bold.

Upate first template	init noise (∓px)	Median	Mean	Std	Max
**✓**	0	1.17	1.90	2.51	24.55
**✗**	16	1.53	2.44	3.18	25.42
**✗**	8	1.45	1.94	2.25	26.45
**✗**	4	1.13	1.69	2.07	20.72
**✗**	2	1.05	1.55	1.60	15.36
**✗**	0	**1.02**	**1.44**	**1.35**	**10.23**

#### 4.3.4 Modality bias

The distribution between Angio and Fluoro varies to some degree in terms of dosage and presence of contrasted vessel structures. We remind the reader that, in our training dataset, the distribution of Angio:Fluoro sequences was 2098:216 of the total of 2314 sequences. Our objective in this study is to develop a model that exhibits strong performance across both modalities. We present the results of training on individual modalities compared with training on combined data in Table 8. Our findings indicate that training solely on one modality results in suboptimal performance on the other modality. Notably, although training on Angio data yields an improvement in Angio performance, training exclusively on Fluoro data fails to enhance performance in Fluoro. We hypothesize that a possible reason for this effect is the imbalance of 2098:216 (Angio to Fluoro sequences), with the following effects.

1. 2098 Angio sequences is a large enough dataset to ensure good Angio performance when training on this data alone;
2. 216 Fluoro sequences is too little to power the training of a large transformer model, leading to inferior results when training/testing on Fluoro only;
3. transitioning from Angio to using all data for training has a negative effect on the Angio test performance—we hypothesize that adding the few Fluoro sequences to training increases the complexity of the training problem, as the distribution of Angio training cases is enhanced with the distribution of Fluoro cases, based on only 216 examples; and
4. transitioning from Fluoro to using all data for training has a positive effect on the Fluoro test performance—we hypothesize that this is because the 216 Fluoro sequences are a complement with many more non-contrasted frames from all Angio sequences to substantially increase the dataset and thereby improve performance.

**Table 8:** Performance variation across modalities based on modality-specific training. The best values are marked in bold.

	Fluoro			Angio			Devices		
Trained on	Mean	Median	Max	Mean	Median	Max	Mean	Median	Max
Fluoro data	1.44	0.84	10.54	4.15	2.36	22.96	6.58	4.62	19.47
Angio data	1.41	**0.75**	11.42	**1.49**	**1.14**	**5.56**	2.80	0.99	22.55
All data	**1.24**	**0.75**	**10.23**	1.61	1.38	7.33	**1.54**	**0.98**	**4.27**

Furthermore, the challenges posed by device obstruction exhibit nuanced differences between Fluoro and Angio, contributing to a reduced performance when the model is trained on a single modality.

## 5 Conclusion

In this study, we presented FIMAE, an MIM approach that is introduced for the purpose of acquiring generalized features from a large unlabeled dataset containing more than 16 million interventional X-ray frames, with the objective of device tracking. FIMAE overcomes the limitation of tube masking as proposed in VideoMAE and applies frame interpolation-based masking for capturing fine inter-frame correspondences. The acquired features are subsequently applied to the task of device tracking within fluoroscopy and angiography image sequences. Our pre-trained FIMAE encoder surpassed all prevalent MIM-based pretraining methods for sequential imaging processing.

The spatio-temporal features acquired during the pretraining phase significantly influenced the extraction and matching of features for the purpose of device tracking. We demonstrated that an efficient spatio-temporal encoder can replace the frequently utilized Siamese-like architecture, yielding a computationally lightweight model that maintains a high degree of precision and robustness in the tracking task. By adopting our methodology, we achieved a noteworthy 23.2% reduction in the maximum tracking error, even without the incorporation of supplementary modules such as flow refinement, when compared with the state-of-the-art multi-modular optimized approach. This performance enhancement was accompanied by a frame-level TSUC score of 97.95% at a 3× faster inference speed than the state-of-the-art method. The results also show that our approach achieved superior tracking performance, particularly in the challenging cases in which occlusions and distractors are present.

### 5.1 Limitations and Future Work

Our investigation is primarily centered on leveraging pre-trained features for the tracking of devices within X-ray sequences. Consequently, we contend that the pre-trained model can be further extended to other tasks within interventional image analytics, such as stenosis detection, guidewire localization, and vessel segmentation. Furthermore, the absence of annotated frames within our sequential imaging dataset imposes a constraint on the utilization of historical trajectory information, a commonly exploited approach in recent single object tracking methodologies in the natural imaging domain. Thus, a more comprehensive investigation is needed to effectively make use of this information in our specific context.

## 6 Appendix A: Pretraining Details

The detailed architecture illustration and the implementation details of the pretraining are illustrated in Tables 9 and 10, respectively. We use a 10-frame vanilla ViT-Base as our encoder architecture; it incorporates joint space-time attention on visible patches. The decoder is of a lower dimension and lower depth than the encoder, which incorporates similar joint space-time attention on all patches. The decoder is only responsible for reconstruction and is discarded for downstream tasks.

**Table 9:** Architecture details of FIMAE. We use a 10-frame vanilla ViT-Base as our architecture. “MHA” here denotes the joint space-time self-attention. The output sizes are denoted by C×T×S for channel, temporal and spatial sizes, respectively.

Stage	Vision transformer (base)	Output size
Data	Temporal gaps = [1,2,3,4]	1 × 10 × 384 × 384
Patch embed	1 × 16 × 16, 768	768 × 10 × 576
	Stride 1 × 16 × 16	
Mask	ρ = tube 75% + frame 98%	768 × 10 × [576 × (1 − ρ)]
Encoder	[MHA(768), MLP(3072)] × 12	768 × 10 × [576 × (1 − ρ)]
Projector	MLP(384) and	
	concat learnable tokens	768 × 10 × 576
Decoder	[MHA(384), MLP(1536)] × 4	384 × 10 × 576
Projector	MLP(256)	256 × 10 × 576
Reshape	from 256 to 1 × 1 × 16 × 16	1 × 10 × 384 × 384

**Table 10:** Pretraining setting.

Config	Name/params
Optimizer	AdamW
Base learning rate	$1.5e^{-4}$
Weight decay	$1e^{-4}$
Optimizer momentum	$\beta_{1}$, $\beta_{2}=0.9$,0.95
Batch size	8
Learning rate schedule	Cosine decay
Warmup epochs	15
Augmentation	MultiScaleCrop

## 7 Appendix B: Downstream Model Details

The architectural detail of the downstream tracking model is depicted in Table 11. The encoder is the same as the pretraining encoder, whereas the decoder is a lightweight transformer decoder, followed by two CNN heads that output the catheter tip heatmap and catheter body mask respectively. The implementation details are further explained in Table 12.

**Table 11:** Architecture details of downstream tracking model. “CA” refers to cross-attention.

Stage	ViT-base + multi-task decoder	Output size
Data	3 templates + 1 search	1 × 3 × 64 × 64, 1 × 1 × 160 × 160
Patch embed	1 × 16 × 16, 768	768 × 148
	Stride 1 × 16 × 16	
	and concatenate	
Encoder	[MHA(768), MLP(3072)] × 12	768 × 148
Projector	MLP(256)	256 × 148
Decoder	Query = (256, 2) and	256 × 148, 256 × 2
	[CA(256), MLP(1024)] × 6	
Cross-correlate	Unconcatenate and matmul	256 × 2 × 10 × 10
Mask head	(Upconv, Conv, Batchnorm, GeLU) × 4	4 × 160 × 160
Mask project	Linear(4,1)	1 × 160 × 160
Heatmap head	(Upconv, Conv, Batchnorm, GeLU) × 4	4 × 160 × 160
Heatmap project	Linear(4,1)	1 × 160 × 160

**Table 12:** Finetuning setting.

Config	Name/params
Optimizer	AdamW
Base learning rate	$6e^{-4}$
Weight decay	$1e^{-4}$
Optimizer momentum	$\beta_{1}$, $\beta_{2}=0.9$,0.95
Batch size	42
Learning rate schedule	Cosine decay
Warmup epochs	10
Augmentation	Horizontal flip
	Vertical flip
	Random rotation (−10 deg, 10 deg)

## Supplementary Material



## Data Availability

Based on the data usage agreements, the data cannot be shared with the community. More information about the code can be shared upon request.
